# The Cuproptosis-Related Long Noncoding RNA Signature Predicts Prognosis and Immune Cell Infiltration in Hepatocellular Carcinoma

**DOI:** 10.1155/2023/9557690

**Published:** 2023-02-27

**Authors:** Ying Li, Kaichao Song, Wensheng Zheng

**Affiliations:** ^1^Beijing City Key Laboratory of Drug Delivery Technology and Novel Formulation, Institute of Materia Medica, Chinese Academy of Medical Sciences & Peking Union Medical College, Beijing 100050, China; ^2^Shandong University of Traditional Chinese Medicine, College of Traditional Chinese Medicine, Jinan 250355, China; ^3^Institute of Medicinal Biotechnology, College of Traditional Chinese Academy of Medical Science and Peking Union Medical College, Beijing 100050, China

## Abstract

**Background:**

Hepatocellular carcinoma (HCC), ranking as one of the most common malignant tumors, is one of the leading causes of cancer death, with a poor prognosis. Cuproptosis, a novel programmed cell death modality that has just been confirmed recently, may play an important role in HCC prognosis. Long noncoding RNA (LncRNA) is a key participant in tumorigenesis and immune responses. It may be of great significance to predict HCC based on cuproptosis genes and their related LncRNA.

**Methods:**

The sample data on HCC patients were obtained from The Cancer Genome Atlas (TCGA) database. Combined with cuproptosis-related genes collected from the literature search, expression analysis was carried out to find cuproptosis genes and their related LncRNAs significantly expressed in HCC. The prognostic model was constructed by least absolute shrinkage and selection operator (LASSO) regression and multivariate Cox regression. The feasibility of these signature LncRNAs used for the evaluation of the overall survival rate in HCC patients as independent factors was investigated. The expression profile of cuproptosis, immune cell infiltration, and the status of somatic mutation were analyzed and compared.

**Results:**

A prognostic model of HCC consisting of seven cuproptosis gene-related LncRNA signatures was constructed. Multiple verification methods have showed that this model can accurately predict the prognosis of HCC patients. It was showed that the classified high-risk group under the risk score of this model had worse survival status, more significant expression of the immune function, and higher mutation frequency. During the analysis, the cuproptosis gene CDKN2A was found to be most closely related to LncRNA DDX11-AS1 in the expression profile of HCC patients.

**Conclusion:**

The cuproptosis-related signature LncRNA in HCC was identified, on the basis of which a model was constructed, and it was verified that it can be used to predict the prognosis of HCC patients. The potential role of these cuproptosis-related signature LncRNAs as new targets for disease therapy in antagonizing HCC development was discussed.

## 1. Introduction

The incidence of liver cancer is increasing year by year [1]. About 1 million people suffer from this disease every year worldwide [2, 3]. Among them, hepatocellular carcinoma (HCC), accounting for 75% to 90%, is the tumor ranking the third in mortality globally [4, 5]. Currently, radiotherapy, chemotherapy, and surgery are often adopted for early HCC, and systemic treatment is used for advanced HCC in clinical practice [6, 7]. However, the early diagnosis of HCC is difficult. Most HCC patients are diagnosed in the late stage, and it is difficult to cure them [8, 9]. Therefore, finding new targets for diagnosis and treatment is crucial for the effective management of HCC.

Cuproptosis is a recently confirmed cell death modality. Copper is one of the essential trace elements for living organisms. It is mainly absorbed through the small intestine and metabolized in the liver and in turn transported to various tissues and organs with the blood for use by cells. Generally, cuproptosis exists widely in body cells in the form of reduced cuprous ions (Cu^+^) and oxidized cupric ions (Cu^2+^), and also it is ingested by the copper transporter (hCtrl) and effluxed by adenosine triphosphatase (ATPase) to its intracellular levels in the body [10, 11]. The homeostasis of copper in the body is associated with many diseases. Existing studies have shown that the destruction of copper homeostasis can lead to the occurrence of multisystem diseases such as anemia [11], Wilson's disease [12], Menkes disease [13, 14], brain disease [15–17], immune system disease [18, 19], and tumors [20, 21]. When the copper ion concentration in the body cells exceeds the threshold of maintaining the homeostasis mechanism, it can directly combine with the fatty acylation components of the tricarboxylic acid cycle, which results in the aggregation of fatty acylated proteins and the loss of iron-sulfur cluster protein, and in turn trigger protein toxic stress and also ultimately cause cell death [22–24].

The long noncoding RNA(LncRNA) is a noncoding RNA with a length of N200 nucleotides. Recent studies have shown that LncRNA plays an important role in the occurrence and development of cancer, and the abnormal expression of LncRNA is associated with malignant tumors, tumor autophagy, tumor resistance, and tumor immunity[25, 26]. So far, it has been found that LncRNA is abnormally expressed in HCC [27], which can play a role in the formation of HCC and the migration of cancer cells, and in turn affect the occurrence, metastasis, and prognosis of HCC. Therefore, many scholars have focused on the value of lncRNA in tumor prognosis, building prognosis models, and developing new diagnostic targets based on the unique expression profile of lncRNA [28, 29]. The development and progression of HCC is a cumulative effect of genetic changes that affect the expression of tumor-related genes [30]. However, as a new mechanism involved in tumor cell death, it is worth paying attention to and discussing whether the influence of the changes of related genes on HCC has far-reaching significance and value.

Therefore, in this study, we used bioinformatics methods, sample data from TCGA public database, combined with cuproptosis-related genes collected by the literature search, to construct a unique expression profile of cuproptosis-related lncRNA, and used machine learning and other methods to screen out lncRNA which is of the great value to the prognosis of HCC and construct a prognosis model. The purpose of this study is to provide more predictive methods for diagnosing and evaluating the prognosis of patients with HCC, and to provide new ideas and support for the development of cuproptosis in HCC.

## 2. Materials and Methods

### 2.1. The Collection of Samples and CRGs

**Figure 1:**
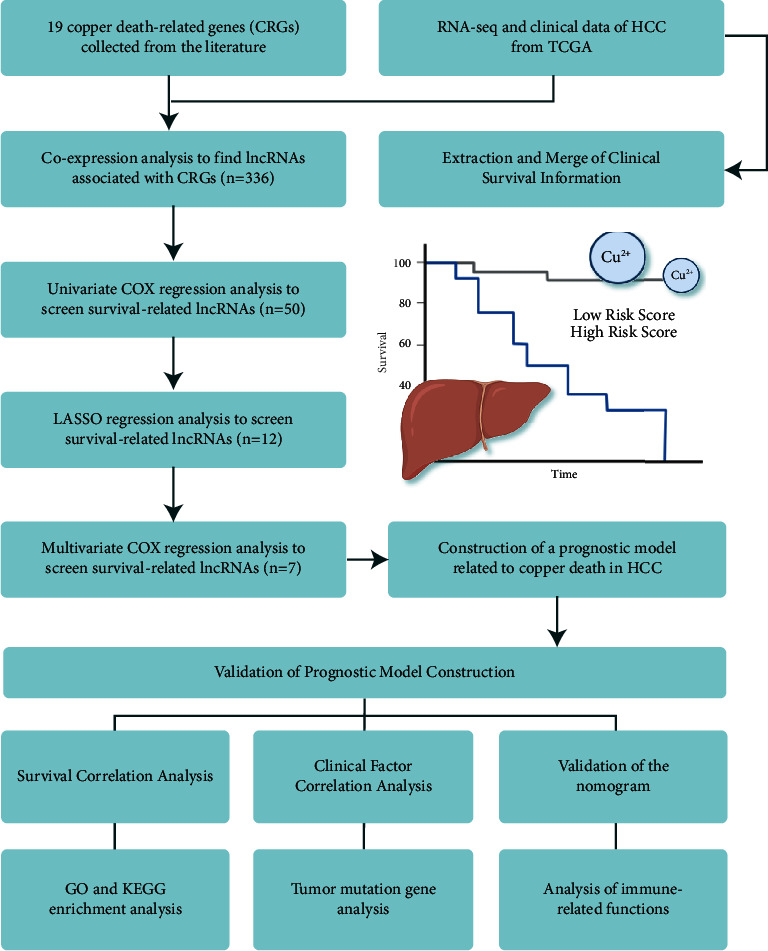
Process flow diagram.

The public data on HCC came from The Cancer Genome Atlas (TCGA) database, including the RNA sequence data, clinical information, and tumor mutation data on 424 patients (50 normal and 374 tumor patients). The R language was used to classify protein-coding genes and lncRNAs in RNA sequences. The clinical information collection included gender, age, stage, grade, TNM, survival time, and status. For the accuracy of the study, the unknown part of clinical information was marked uniformly. A total of 19 CRGs were collected through the literature search. The research process is shown in Figure 1.

### 2.2. Coexpression Analysis of Cuproptosis-Related LncRNAs

The “limma” package in R language was used to extract the expression levels of CRGs and LncRNA from HCC samples. The LncRNAs associated with these CRGs were obtained by coexpression analysis, and also their relationship with HCC was tested by correlation analysis. The absolute value of the correlation coefficient was set to >0.5 and *p* value <0.001(*p* < 0.001). The Sankey diagram is a diagram used to describe the flow direction of values from one group to another. In order to more intuitively show the relationship between CRGs and its related lncRNA, we used dplyr, ggalluvial, and ggplot2 packages in R language to draw the Sankey diagram to visualize the coexpression results.

### 2.3. Screening and Construction of a Prognostic Cuproptosis-Related lncRNAs Signature

The expression of lncRNA related to cuproptosis in the same sample of patients with HCC was combined with their survival status and survival time data. The lncRNAs related to cuproptosis was randomly divided into a training set (the train group) and a testing set (the test group) (*n* = 1) by the random forest algorithm, and the ratio of the training set to testing set was set to 1 : 1. The cuproptosis-related lncRNAs were filtered using univariate Cox regression analysis for genes that significantly affected the overall survival of patients with HCC (*p*-value <0.05). The univariate COX results were screened using a least absolute shrinkage and selection operators (LASSO) regression analysis. The multivariate COX regression analysis can detect whether multiple features are related to survival at the same time. In order to make the screened prognosis lncRNAs results more accurate, we further screened it by using multivariate COX regression based on the results of LASSO regression analysis. The screening result was a prognostic lncRNA associated with cuproptosis in HCC. We also constructed a prognostic model based on these key lncRNAs for evaluating the prognostic survival of patients with HCC. The model calculation formula is as follows:

(1) $$\begin{array}{c} risk\;score=\overset{n}{\underset{i=1}{\sum }}\beta_{i}\times x_{i}\text{.} \end{array}$$

*β*_*i*_ represents the regression coefficient of each LncRNA, and *X*_*i*_ the expression level of each LncRNA.

The risk scores of the train and test groups were predicted according to the model, and the samples were divided into the high- and low-risk groups according to the median value of the risk scores.

### 2.4. Survival and Independent Prognostic Analyses

The survival analysis of patients in high- and low-risk groups was performed by models. Progression-free-survival (PFS) is an important index commonly used in clinical practice to judge the survival of malignant tumor patients in addition to OS. It represents the time period from the initial treatment of cancer patients to disease progression or death. The longer the PFS time is, the longer the survival cycle of this patient will be. In this study, the survival analysis was separately performed from two aspects of OS and PFS to evaluate the value of these cuproptosis-related LncRNA markers in HCC survival and prognosis.

Through independent prognostic analysis, it is possible to observe whether the prediction model constructed by us can be used as an independent prognostic factor independently of other clinical traits. The unifactor analysis was adopted to compare each clinical factor with survival time and status, and also the multifactor analysis of the effect on the survival status was analyzed under the interaction of multiple factors. The correlation between CRGs and prognosis-related LncRNA was verified, with the correlation heat map.

In order to verify the accuracy of the prediction model constructed by us, the receiver operating characteristic (ROC) curve of 1-, 3-, and 5-year survival in HCC patients under the prediction of this model and that under the prediction of it combined with other clinical traits were plotted, respectively. The prediction accuracy of this model was confirmed by interpreting the area under the curve (AUC). Additionally, the concordance index (C-index) curve was plotted to evaluate the probability of consistency between the predicted and actual results. Principal component analysis (PCA) is a multivariate statistical method that can help us to evaluate the expression differences of four variables: whole genes, CRGS, CRGs-related lncRNAs, and characteristic lncRNAs for constructing models.

### 2.5. The Construction of a Prediction Nomogram

Based on the multifactor regression analysis, scores were assigned to each clinical factor according to the effect of various clinical traits in the prediction model on OS. The 1-, 3-, and 5-year OS of HCC patients was estimated through the relationship between the total score and the probability of outcome event occurrence and displayed in the form of a nomogram.

### 2.6. Model Validation for Clinical Grouping

By grouping clinical information, it was verified whether our prediction model is suitable for patients with different clinical straits from three aspects of tumor: grade, stage and age. According to the degree of tumor differentiation, G1-G2 patients with a relatively low malignant degree of HCC were grouped into one group, and G3-G4 patients with a high malignant degree were grouped into one group; the early patients in stages I and II were grouped into a group, and the late patients in stage III and IV were grouped one group; they were divided into two groups according to the age of <65 and ≥65, and the survival curve was plotted.

### 2.7. Risk Differentially Expressed Genes and Their Functional Enrichment Analysis

Through the “limma” package, the gene expression levels of high- and low-risk groups from the sample data were extracted and compared to find the genes with the differential expression between high- and low-risk groups (logFC >1, FDR >0.05). These risks differentially expressed genes may provide new ideas for explaining the HCC progression. Through gene ontology (GO) analysis, these risk differentially expressed genes that are involved in the biological processes of the body could be enriched and found. The biological processes included three aspects, the biological function (BP), cell component (CC), and molecular function (MF). Through the analysis of the Kyoto Encyclopedia of Genes and Genomes (KEGG), the signal pathways related to these differentially expressed genes could be enriched and found.

### 2.8. Tumor Mutation Analysis

By sorting and analyzing the mutation information in high- and low-risk groups, the genes mutated in the two groups of samples and their mutation frequency could be obtained. Also, the differential analysis of tumor mutation burden between the two groups of HCC patients was performed. It was observed whether the tumor mutations between the two groups were significantly different, and the 15 genes with the highest mutation frequency in HCC were further found for visualization, observation, and interpretation.

### 2.9. Analysis of the Immune-Related Function

Through gene set variation analysis (GSVA), the immune-related function gene set enriched by a single gene can be used as a signature expression matrix. Also, the difference in the immune function between the two groups by comparing the difference in the gene set expression between high- and low-risk groups was inferred. The difference analysis of immune checkpoint genes can help us observe which immune checkpoint genes are different in high- and low-risk patients to further find the relationship between cuproptosis genes and immune checkpoints.

## 3. Research Results

### 3.1. The Consistency Test of Clinical Traits in Patients between the Train and Test Group Cohorts

According to the clinical information, the consistency test on the clinical traits of patients in the train and test groups was carried out. The missing unknown parts of the clinical information were uniformly marked with “unknown.” A total of 370 HCC patients were included. The test results are shown in Table 1. It was seen that after the included sample data were randomly assigned to two groups of cohorts, there was no significant difference in clinical traits between the two groups (*p* > 0.05), with the better-randomized grouping.

### 3.2. The Screening of Cuproptosis-Related LncRNAs and Prognosis-Related LncRNAs in HCC Patients

**Figure 2:**
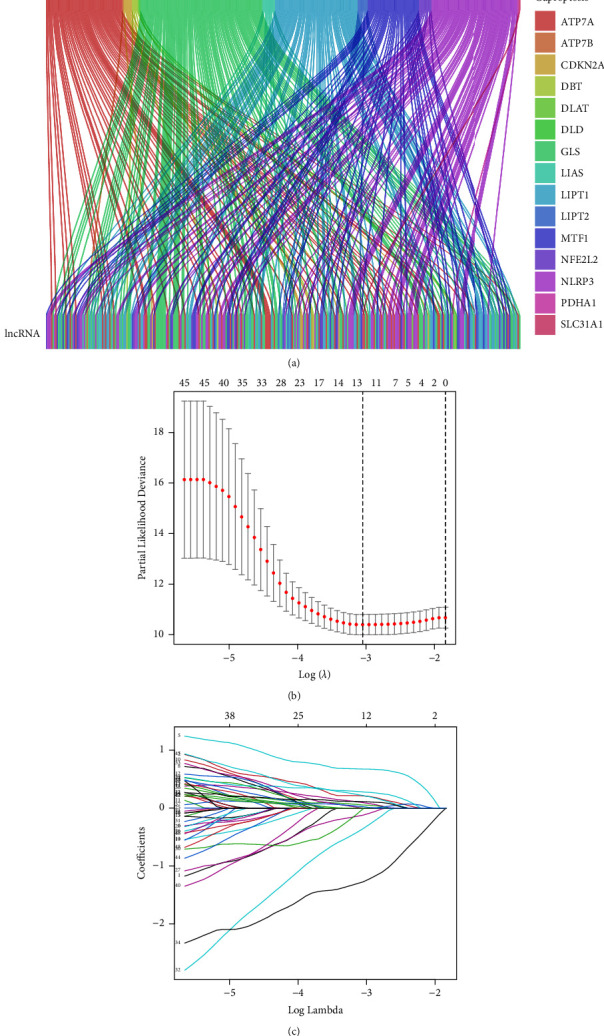
(a) Sankey diagram of CRGs and its related lncRNA: the different colors on the right side of the picture represent different CRGs, the top of the picture represents CRGs, and the bottom represents the lncRNA related to cuproptosis. (b) Univariate cox regression analysis forest plot. (c, d) LASSO regression analysis and cross-validation results. The dotted line on the left in (c) shows the number of features with the smallest error. (d) The changing track of each independent variable coefficient.

**Figure 3:**
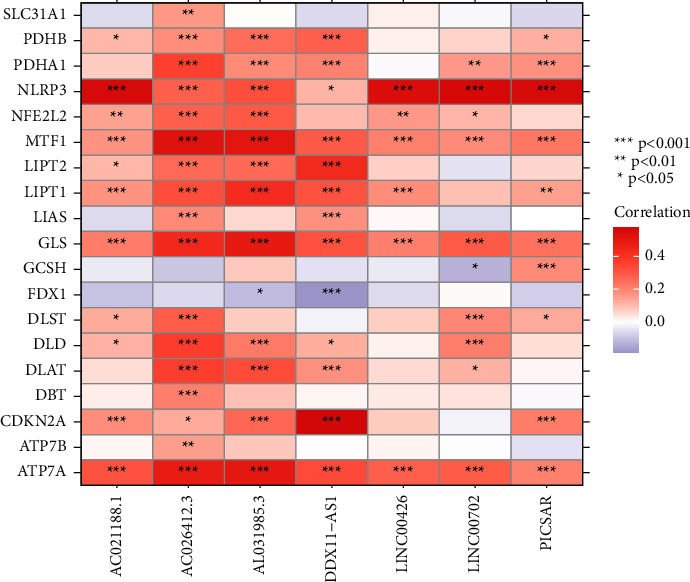
The expression heat map of cuproptosis-related genes (CRGs) and their related long noncoding RNAs (LncRNAs). The right side of the map represents different cuproptosis-related genes (CRGs), the lower part of the map represents the cuproptosis gene-related signature long noncoding RNAs (LncRNAs) screened by least absolute shrinkage and selection operator (LASSO) and multifactor regression, and the color represents the correlation between the two. The closer the color is to red, the higher the correlation is, and the closer it is to purple, the lower the correlation is.

The biomarkers play an important role in tumor detection and treatment. The risk stratification for screening can be increased by finding new biomarkers that may identify susceptibility or early stages of the disease, either alone or as a complement to existing tests. [15, 30] The cuproptosis-related LncRNAs predicted by coexpression were screened according to the correlation coefficient (>0.5), and finally, 15 CRGs and 336 LncRNAs related to them were obtained (Table S1 and Figure 2(a)). After merging the survival information, 50 lncRNAs with the significant correlation and the overall survival rate of patients were obtained by univariate Cox regression analysis H, and the forest plot of survival results was drawn thereby (Figure 2(b)). The 12 features with the smallest error from the 50 significant lncRNs were screened out by LASSO regression analysis as key lncRNAs (see Figures 2(c) and 2(d)). Finally, further screening by multivariate Cox regression identified seven cuproptosis-related prognostic lncRNAs (lncRNA AC026412.3, PICSAR, AC021188.1, LINC00702, LINC00426, AL031985.3, and DDX11-AS1). The risk score was calculated for the prognostic model construction according to the formula in the Section 2. According to the median value of the risk score, the samples were divided into 191 cases in the high-risk group (92 cases in the train group and 99 cases in the test group) and 179 cases in the low-risk group (93 cases in the train group and 86 cases in the test group). The correlation analysis between CRGs and their related signature LncRNAs showed that there was a significant positive correlation between cyclin-dependent kinase inhibitor 2A (CDKN2A) and LncRNA DDX11-AS1, and a close positive correlation between NACHT, LRR, and PYD domains-containing protein 3 (NLRP3) and the LncRNAs(AC02118.1, LINC00426, LINC00702, and PICSAR), while there was a close negative correlation between FDX1 and LncRNA DDX11-AS1 (Figure 3).

### 3.3. The Survival Outcome and Multifactor Test

**Figure 4:**
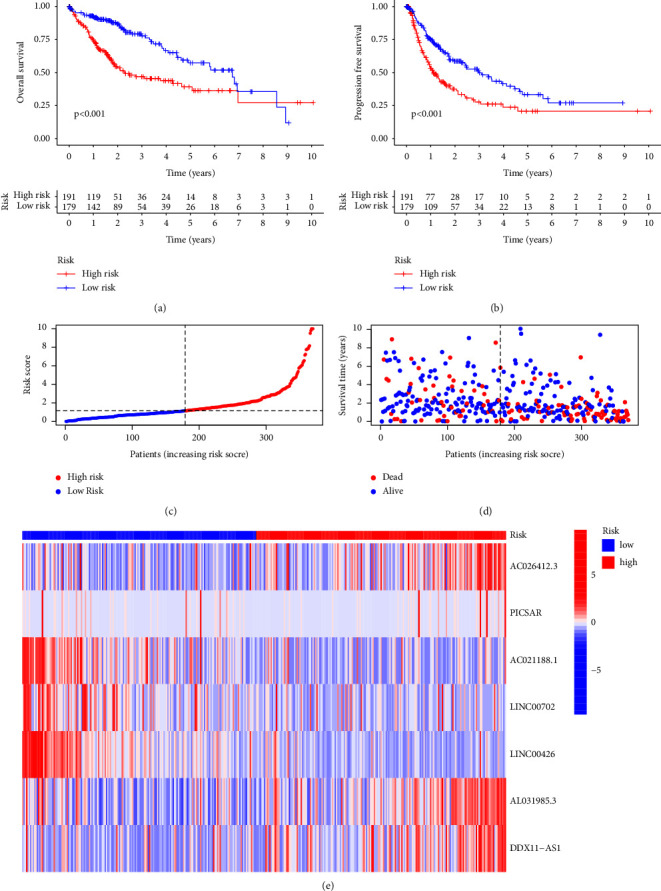
The group diagram of survival outcomes. (a) The Kaplan–Meier curve of overall survival (OS) in high- and low-risk groups; (b) the Kaplan–Meier curve of PFS in high- and low-risk groups; (c) the schematic diagram of the high- and low-risk distribution of hepatocellular carcinoma (HCC) patients; (d) the scatter plot of survival status of high- and low-risk groups; (e) the expression heat map of prognosis-related long noncoding RNA(LncRNA) in high- and low-risk groups.

The survival curve can show the difference in model prediction of patient OS between the high- and low-risk groups. The OS of HCC patients in the high-risk group was generally lower within ten years, but after ten years, the OS was more stable than that in the low-risk group (Figure 4(a)). PFS analysis shows that the significant expression of prognosis-related lncRNA in the high-risk group is related to the poor survival of patients (*P* < 0.001), as shown in Figure 4(b). Additionally, by the risk distribution of HCC patients and the corresponding survival status chart, it was found that the survival status of patients in the high-risk group was worse and their survival time was shorter than that in the low-risk group (Figures 4(c) and 4(d)). By the heat map of the prognostic LncRNA expression in high- and low-risk groups, it was found that the LncRNAs(AC026412.3, AL031985.3, and DDX11-AS1) were positively correlated with the risk score, while the LncRNAs (AC021188.1, LINC00702, and LINC00426) were negatively correlated with the risk score (Figure 4(e)).

**Figure 5:**
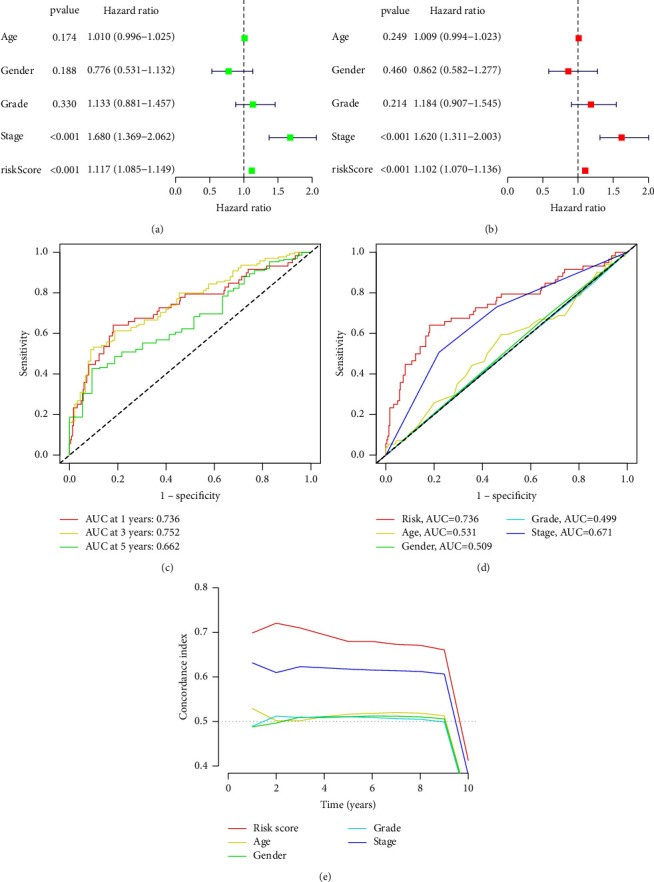
The group diagram for testing prognostic models. (a) The forest diagram of unifactor analysis of independent prognosis; (b) the forest diagram of multifactor analysis of independent prognosis; (c) the time-dependent receiver operating characteristic (ROC) curve diagram for validation of prognostic model risk scores; (d) the receiver operating characteristic (ROC) curve diagram of the accuracy of prediction ability of each clinical trait; and (e) the C-index curve diagram of the accuracy of prediction ability of each clinical trait.

The forest diagram of unifactor and multifactor analyses of independent prognosis showed that the prediction model constructed by us could be used as an independent factor to evaluate the HCC prognosis just like the tumor grade (Figures 5(a) and 5(b); *p* < 0.001). Both the ROC curve and the area under curve (AUC) showed that this prediction model had relatively high accuracy in predicting the prognosis and survival of patients, which is significantly better than the prediction ability of other clinical traits (Figure 5(d)). The time-dependent receiver operating characteristic (ROC) and C-index curves also showed that this model had a good prediction effect, with more sensitivity in the prediction of early HCC patients (Figures 5(c) and 5(e)).

**Figure 6:**
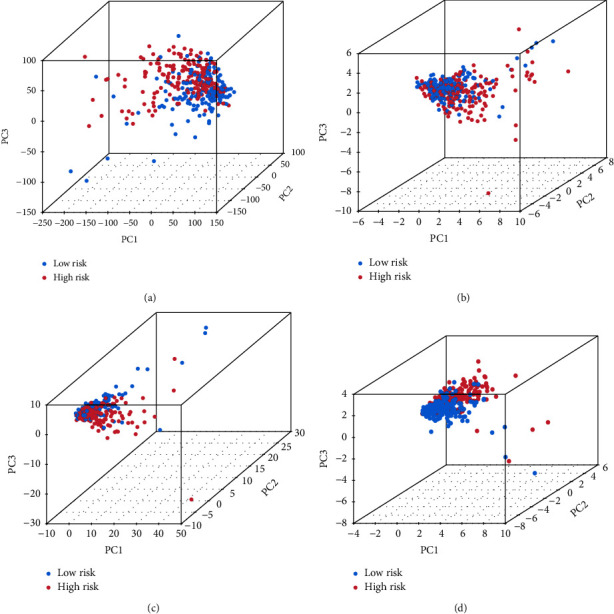
The group diagram of principal component analysis (PCA). (a) The whole gene expression in hepatocellular carcinoma (HCC) patients in two high- and low-risk groups; (b) the expression of cuproptosis genes; (c) the expression of cuproptosis-related long noncoding RNAs (LncRNAs); and (d) the expression of signature long noncoding RNAs(LncRNAs) for model construction.

Through principal component analysis (PCA), the ability of cuproptosis genes, cuproptosis-related LncRNAs, and the signature LncRNAs used for model construction in classifying high- and low-risk patients could be judged. The results showed that the classification ability of signature LncRNAs was much better (Figure 6(d)), which demonstrated that it has a good value in prognostic risk prediction of hepatocellular carcinoma(HCC) patients.

### 3.4. The Nomogram of Prognostic Models

**Figure 7:**
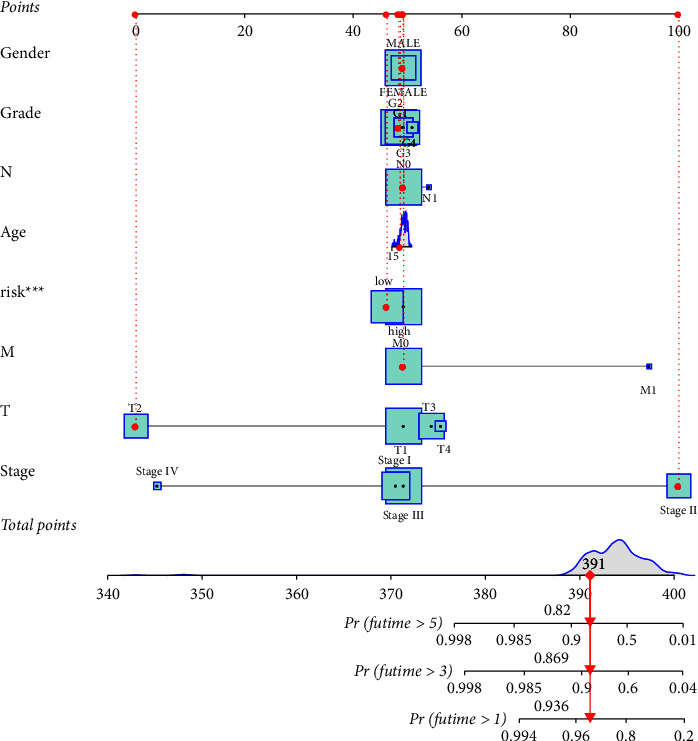
The nomogram of each clinical trait and prognostic cuproptosis-related long noncoding RNA (LncRNA).

Based on the constructed prediction model based on the cuproptosis-related signature LncRNA and the effects of each clinical trait in the model on HCC, a nomogram was made to evaluate the prognosis of HCC patients for clinical management of HCC patients. The ordinate of the nomogram represents the variables in the prediction model and the abscissa represents the range of values that can be taken for each variable. According to the number of the sample data above the vertical corresponding scale point on the horizontal axis of the variable, the score of the single variable of the sample can be obtained. The vertically correspond survival probability value can be found by calculating that total score of variable, thereby judging the survival condition of the patient. One patient sample was randomly selected from the samples for prediction, and its 1-, 3-and 5-year prediction scores are shown in Figure 7. The model showed that the total score of the patient was 391, and the survival probability was 93.6% after one year, 89.6% after three years, and 82.0% after five years after the diagnosis of HCC.

### 3.5. Clinical Grouping for Model Verification

**Figure 8:**
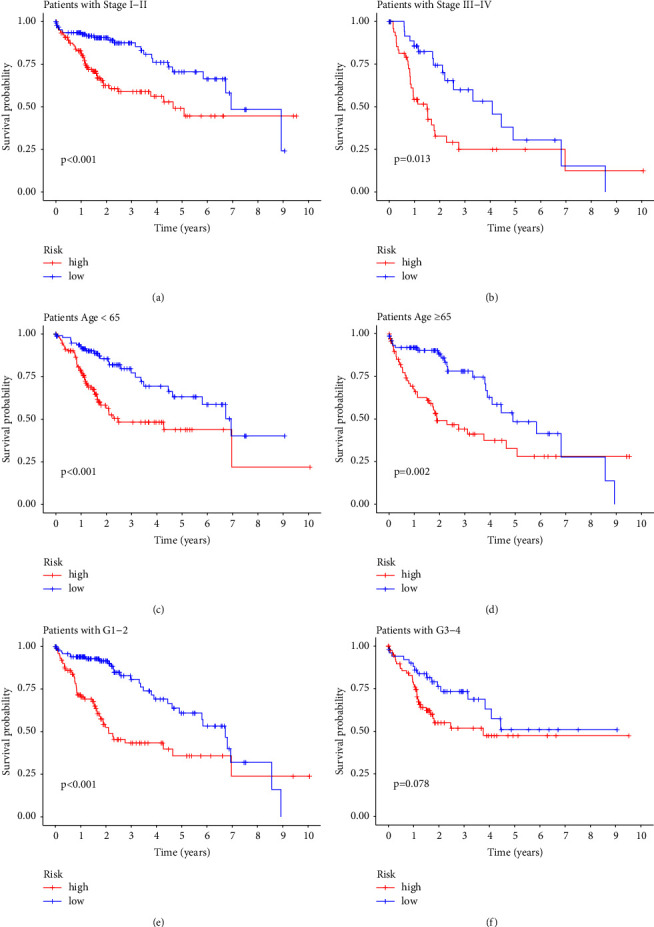
The Kaplan–Meier curve of verification of model-predicted overall survival by grouping according to clinical trait. (a, b) the survival analysis curve of high- and low-risk groups grouped according to the tumor stage, I-II (early stage) and III-IV (latestage). (c, d) the survival analysis curve of high- and low-risk groups grouped according to age. (e, f) the survival analysis curve of high- and low-risk groups grouped according to tumor differentiation levels. G1-G2 represent medium and high differentiation levels, with low malignancy, and G3-G4 represent low or undifferentiated levels, with high malignancy.

As shown in Figure 5, this prediction model can be used as an independent factor to evaluate the prognosis of HCC and has better prediction ability compared with tumor staging. The samples were grouped according to the clinical information, and the performance of the prognostic model was verified by grouping in terms of stage, age, and grade. According to the clinical information, the samples were separately grouped from three aspects of grade, stage, and age to verify the accuracy of the model in predicting the prognosis of patients. In the survival curve drawn according to tumor stage factors, it can be seen that the patients in the high-risk group divided by the model showed worse survival status in both early and late stages of the tumor (Figures 8(a) and 8(b)). The same situation also appeared in the survival curves obtained by age grouping (Figures 8(c) and 8(d)). However, the results of grouping verification according to tumor differentiation levels were slightly different. According to the results of the C-index curve, we can find that the tumor grade (grade) has poor ability to judge the prognosis and survival of patients with HCC. Patients with high- and low-risk scores showed significant differences between the G1-G2 groups (*P*  <  0.001), as shown in Figure 8(e). However, among the more differentiated G3-G4 groups, there was no significant difference in survival condition between the high- and low-risk groups (*P*  >  0.05). The Kaplan–Meier curve shows that our model still has good discrimination ability in the early stage of the disease in patients with poorly differentiated HCC, as shown in Figure 8(f). However, it lacks sensitivity in the long-term prediction in patients with poorly differentiated HCC. We believe that although the public database contains data on all stages of the tumor, the prognosis of HCC patients with low differentiation and high malignancy is often worse, and their long-term survival performance is unoptimistic. Therefore, there is a lack of long-term survival data from poorly differentiated patients, resulting in a mediocre performance of the model in patients during this period. This suggests that in order to increase the credibility of the model, more sample data should be included in future research.

### 3.6. Analysis of Risk-Differential Genes and Their Functional Enrichment

**Figure 9:**
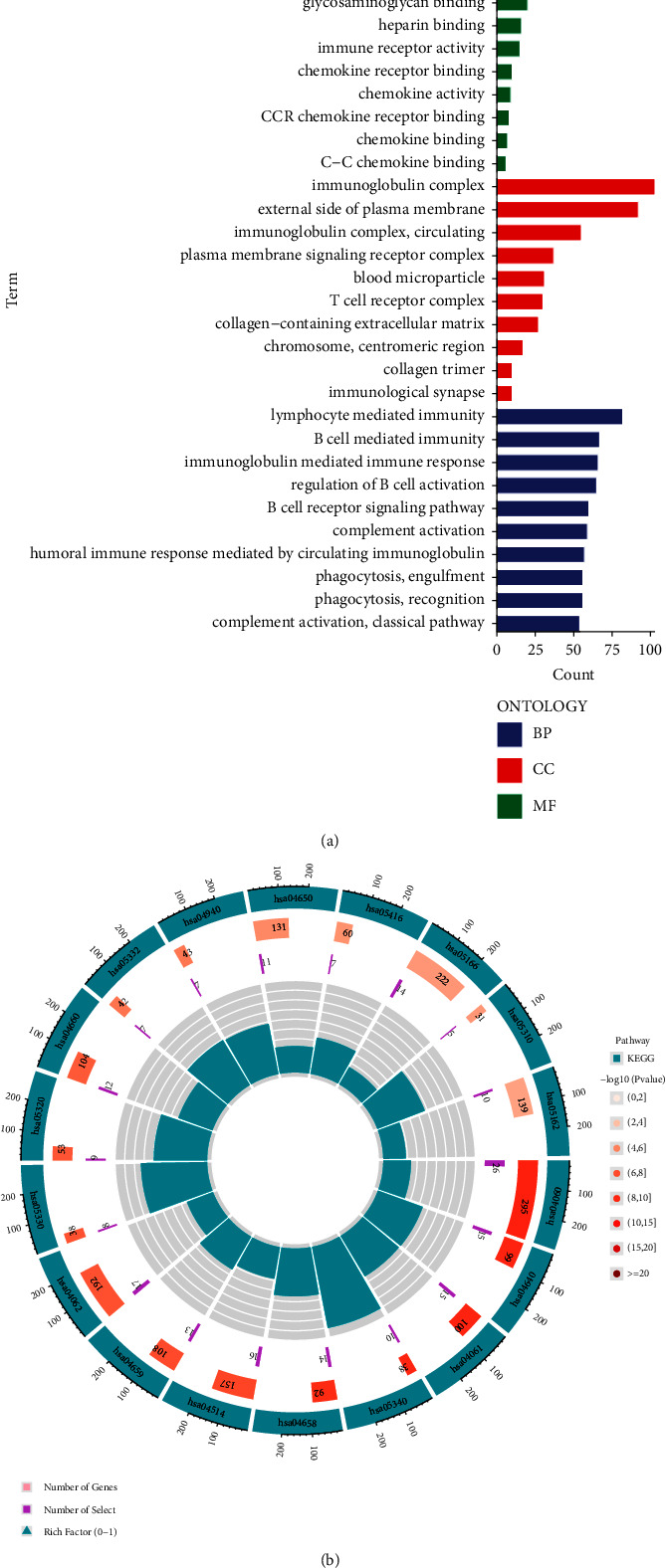
The enrichment analysis diagram of differential genes in high- and low-risk groups. (a) The color bar diagram of gene ontology (GO) analysis of risk differential genes, with colors representing biological function (BP, in BLUE), cell component (CC, in RED), and molecular function (MF, in GREEN), respectively (b) the circle diagram of Kyoto Encyclopedia of Genes and Genomes (KEGG) pathway enrichment analysis of risk differential genes, in which cyan represents the pathway number, different degrees of red represents significance, and purple represents the number of enriched target genes.

Through risk differential analysis, a total of 598 genes with the differential expression were screened in high- and low-risk groups. The top ten with the highest significance among the gene ontology (GO) enrichment analysis results were selected and sorted according to the number of target genes enriched in them, and they were integrated into a classification histogram (Figure 9(a)). As can be seen in the figures, in BP, these differential genes were mainly involved in these biological functions such as lymphocyte-mediated immunity, B cell-mediated immunity, immunoglobulin-mediated immune responses, regulation of B cell activation, the B cell receptor signaling pathway, complement activation, humoral immune response mediated by circulating immunoglobulin, phagocytosis recognition, and complement activation of the classical pathways; in CC, cell components such as the immunoglobulin complex, external side of the plasma membrane and the plasma membrane signaling receptor complex, and blood microparticles were involved; in MF, these risk differentially expressed genes were mainly involved in molecular functions such as antigen binding, immunoglobulin receptor binding, glycosaminoglycan binding, heparin-binding, immune receptor activity, chemokine receptor binding, chemokine activity, CCR chemokine receptor binding, chemokine binding, and C–C chemokine binding.

Kyoto Encyclopedia of Genes and Genomes (KEGG) pathway enrichment analysis showed that the pathways closely related to these risk differentially expressed genes included those such as hsa04060 cytokine-cytokine receptor interaction, hsa04640 hematopoietic cell lineage, hsa04061 viral protein interaction with cytokine, and cytokine and hsa05340 primary immunodeficiency (Figure 9(b); supplementary Table S2).

### 3.7. Mutation Gene Analysis

**Figure 10:**
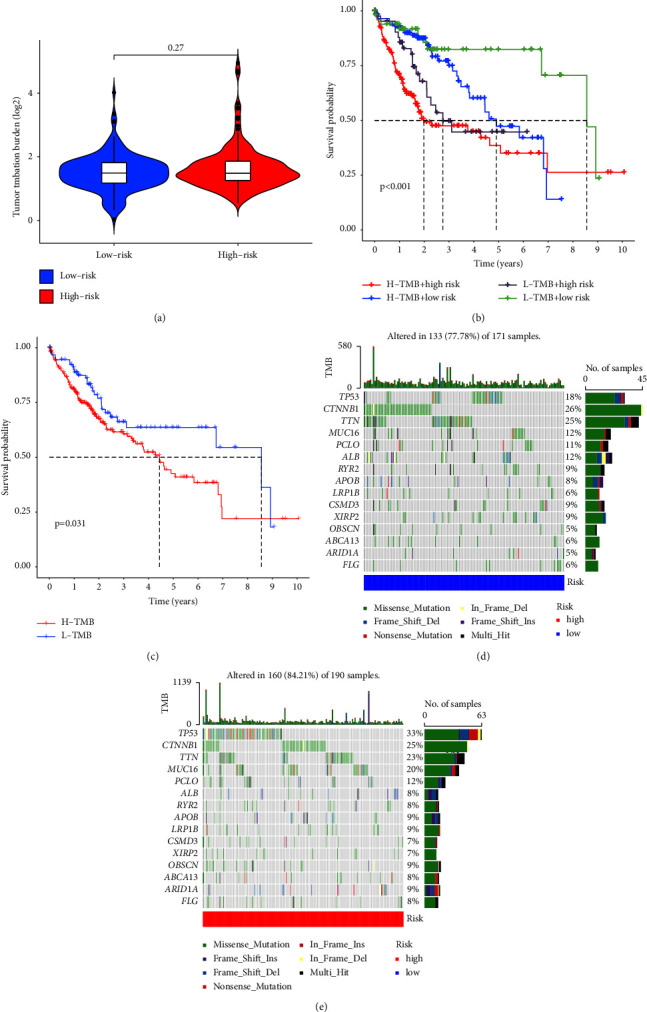
The group diagram of analysis of immune-related functions and mutated genes. (a) The violin diagram of mutation burden comparison in the high- and low-risk groups of hepatocellular carcinoma (HCC) patients; (b) the Kaplan–Meier curve for survival analysis of high and low mutation burden groups; (c) the Kaplan–Meier curve for survival analysis of high and low mutation burden groups combined with high- and low-risk groups; (d, e) the waterfall diagram of the mutation frequency of HCC tumors in both low- and high-risk groups, which shows the 15 genes with the highest mutation frequency in HCC patients, with different colors representing the mutation method of this gene.

After analyzing mutation information between the high-risk group and the low-risk group, we found that there was no significant difference in the tumor mutation load between these two groups, which meant that the difference in the number of gene mutation sites in HCC cells between these two groups was insignificant, as shown in Figure 10(a). The survival analysis was performed according to the mutation burden, and it can be seen from Figure 10(b) that the survival status of patients in the high burden group was worse (*P*  <  0.05). From the survival analysis results based on tumor mutation burden combined with the patient risk, it can be seen that there were significant differences between all the four groups (*P*  <  0.001), and the patients in the high-risk group all showed a poor survival status regardless of the high or low level of the tumor mutation burden. This indicates from the side that the prediction model we constructed has a certain value in the analysis of patient survival prognosis (Figure 10(c)).

The waterfall plot of the two groups of mutated genes shows that they had a higher mutation frequency in the high-risk group. Among them, the genes such as TP53 (cellular tumor antigen p53), CTNNB1 (catenin beta-1), TTN (titin), and MUC16 (mucin-16) had a very high mutation rate in HCC, with their mutation methods all mainly missense mutation, and some genes were also mutated by such methods as frame shift mutation and nonsense mutation. Among them, the variation frequency of GTNNB1, TTN, and MUC16 in the low-risk group was higher than that in the high-risk group, and on the contrary, TP53 is more prone to mutation in the high-risk group (Figures 10(d) and 10(e)).

### 3.8. The Analysis of Immune-Related Functions

**Figure 11:**
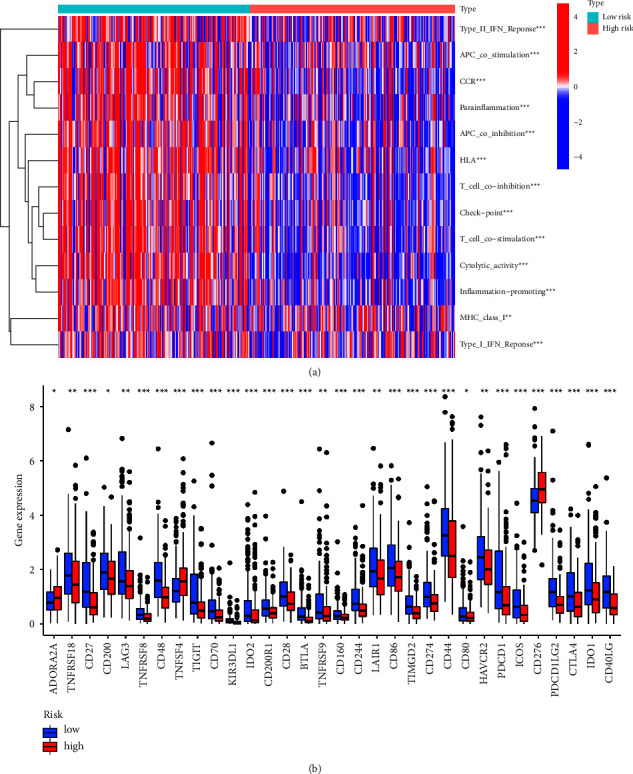
The group diagram of analysis of the immune-related function and immune checkpoint gene. (a) The expression heat map of 13 immune-related gene sets in high- and low-risk groups and (b) the box plot of differential analysis of immune checkpoint genes in patients of high- and low-risk groups.

**Figure 12:**
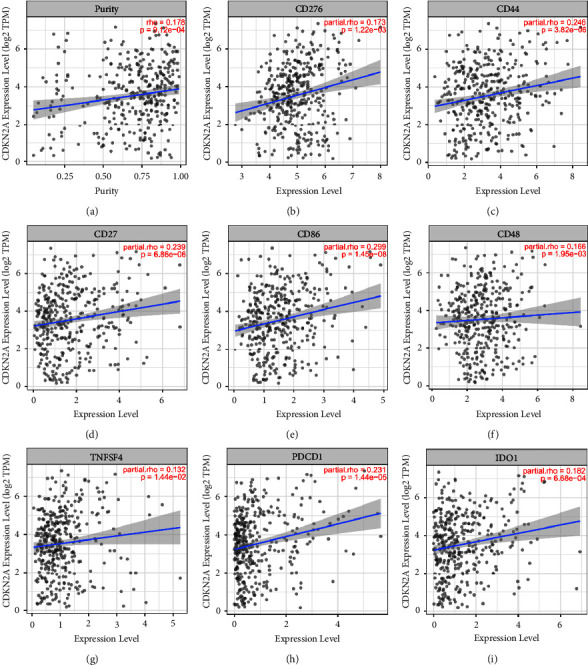
The scatter plot of correlation between the key cuproptosis gene CDKN2A and immune checkpoint genes with significant expression differences.

Tumor immunotherapy is considered as a promising method for tumor treatment, and it has become an important method and research focus of tumor treatment [31, 32]. Through the analysis of immune-related functions, it could be found that the expression of these 13 immune-related functions was all significantly different between high- and low-risk groups (*P*  <  0.01), and they consistently showed a negative correlation in the high-risk groups (Figure 11(a)). Due to the importance of checkpoint inhibitor-based immunotherapy, the differences in the immune checkpoint gene expression in high- and low-risk groups of HCC patients were investigated (Figure 11(b)). The 25 immune checkpoint genes such as CD276, CD44, PDCD1, and TNFSF4 were closely related to HCC (*P*  <  0.001). Combined with the expression of cuproptosis genes in HCC (Figure 12), CDKN2A, the most significantly expressed cuproptosis gene, taken as an example, the correlation between it and 8 immune checkpoint genes was explored and also a scatter plot was drawn (Figure 11). The blue line in the figure represents the trend of the correlation between CDKN2A and the corresponding immune checkpoint genes, indicating that all eight immune checkpoint genes were positively correlated with CDKN2A.

## 4. Discussion

Although cuproptosis was formally put forward as a newly defined programmed cell death [24], the role of copper in body cells has long been mentioned in studies by many scholars. Copper can induce various forms of cell death through various mechanisms, including apoptosis and autophagy [33] and can play a role of interfering with the progression of tumors and improving the therapeutic effect in tumors [20, 21, 34–36]. Studies have showed that tumor cells have a higher demand for copper than normal cells [37]. This phenomenon has been confirmed at the sites of many tumors, including breast cancer [38, 39], lung cancer [40], gastrointestinal tumors [41, 42], and oral cancer [43, 44]. Copper can affect the vascular endothelial growth factor [45, 46], fibroblast growth factor [47], and tumor necrosis factor [48] and also promote angiogenesis, which is conducive to the occurrence, development, and metastasis of tumors. However, when the concentration of copper at the tumor site is abnormal, copper can regulate autophagy through ULK1 and ULK2 [49], and control protein quality through UBE2D2, which in turn affects the growth and progression of the tumor [49–51].

Therefore, in this study, bioinformatic methods were used to explore the potential role of copper and its CRGs in HCC from the perspective of cuproptosis. The expression of CRGs in HCC was determined, and 15 target genes with the significant correlation were obtained by screening. The expression levels of all LncRNAs in HCC samples were extracted, and 336 LncRNAs related to these CRGs were found through coexpression analysis. Univariate Cox regression, LASSO regression analyses, and multivariate Cox regression analysis were used to further screen out the LncRNAs (AC026412.3, AL031985.3, DDX11-AS1, AC021188.1, LINC00702, and LINC00426) with the signature expression. A prognostic model consisting of these signature LncRNAs was further constructed.

In order to verify the applicability and accuracy of this prognostic model, the sample patients were divided into high- and low-risk groups according to the risk score. By evaluating the survival status of patients in the high and low risk groups divided by the model, we can verify the clinical predictive ability of the model and compare the differences in immune function and tumor mutations between the two groups of patients. In the analysis of survival results of the high-risk group and the low-risk group, OS shows that the low-risk group performs better in the early years, and we think that this result is related to the insufficient sample size. HCC, as a malignant tumor with high mortality, is often found to be advanced, and because of the difficulty in treatment, the prognosis of patients is often unsatisfactory. It is found that the average life span of patients with HCC after diagnosis is only 4.9 years [52]. In order to verify the value of the prognostic model in survival, we supplemented PFS analysis. It was confirmed that this model with a good predicative ability can not only accurately predict the survival status of HCC patients, but also is more sensitive in the prediction of early HCC patients. Liu et al. have also paid attention to the significance of cuproptosis-related genes in the prognosis of liver cancer in their research, and their research samples are the same as ours. However, their research pays more attention to the analysis of cuproptosis-related immune functions, hypoxia-related genes, and tumor mutation load and drug sensitivity. Nevertheless, their research results are consistent with ours, which can prove the reliability of our conclusion to a certain extent [28].

During the analysis, it was found that the expression of the cuproptosis gene CDKN2A was the most significant in HCC and that it was closely and positively correlated with LncRNA DDX11-AS1. Studies have showed that CDKN2A is significantly expressed in multiple cancer tissues, thus affecting the prognosis of a variety of cancers. CDKN2A is negatively correlated with serosal invasion in the cervical cancer tissue [53]. Also, it can promote the angiogenic phenotype of esophageal squamous cell carcinoma and predict a poor prognosis [54]. Luo et al. reported that there is a certain correlation between the CDKN2A expression as well as immune invasion and the risk of HCC occurrence and that the high expression of CDKN2A is negatively correlated with the overall survival rate and prognosis of patients [55], which may be related to the participation of CDKN2A in the MAPK signaling pathway and the diversity of liver cancer [56]. Additionally, Luo et al. believed that the expression of CDKN2A may help to regulate tumor-related macrophages, dendritic cells, and T cells and that CDKN2A may play an important role in immune infiltrating cells and also can be used as one of the prognostic biomarkers of HCC patients [55].

DDX11-AS1 is a newly discovered LncRNA, which is abnormally highly expressed in multiple malignant tumors [57], such as HCC, colorectal cancer, and gastric cancer. DDX11-AS1 plays its carcinogenic role by regulating the expression of related genes directly or indirectly, with the following examples given: DDX11-AS1 can bind to HNRNPC to promote the proliferation and migration of glioma cells [58] and silencing DDX11-AS1 can inhibit the growth of HCC cells by upregulating TRAF5 [59]. These results have suggested that DDX11-AS1 may play a significant regulatory role in tumors. So far, there has been no research on the relationship between CDKN2A and DDX11-AS1. We boldly inferred that there may be a positive regulatory relationship between CDKN2A and DDX11-AS1, and that silencing DDX11-AS1 can indirectly inhibit the CDKN2A expression, thereby increasing the role of copper loading in tumor cells, promoting cuproptosis, and increasing tumor cell apoptosis. However, more in-depth research and practices are still required for proving whether the fact is as we speculated.

In conclusion, a cuproptosis-related LncRNA model was constructed, which can be used for the prediction of HCC prognosis. However, there are also some limitations of this study. First, due to the currently incomplete understanding of cuproptosis, in this study, there was no guarantee that all landmark components were only related to cuproptosis, and the specific role of cuproptosis in HCC could not be independently assessed. Additionally, the prognostic ability of cuproptosis-related LncRNA in HCC was made statistically and analyzed only through the samples in the database, but it still needs the support of massive clinical data and the verification of basic research. Nevertheless, we believe that this study may provide more ideas for improving the prognosis prediction of HCC patients.

## Figures and Tables

**Table 1:** The consistency test results of HCC clinical traits.

Covariates	Types	Total	Test group	Train group	*p* value
Age	≤65	232 (62.7%)	107 (57.84%)	125 (67.57%)	0.0676
	>65	138 (37.3%)	78 (42.16%)	60 (32.43%)	

Gender	Female	121 (32.7%)	60 (32.43%)	61 (32.97%)	1
	Male	249 (67.3%)	125 (67.57%)	124 (67.03%)	

Grades	G1	55 (14.86%)	24 (12.97%)	31 (16.76%)	0.7163
	G2	177 (47.84%)	93 (50.27%)	84 (45.41%)	
	G3	121 (32.7%)	60 (32.43%)	61 (32.97%)	
	G4	12 (3.24%)	6 (3.24%)	6 (3.24%)	
	Unknow	5 (1.35%)	2 (1.08%)	3 (1.62%)	

Stages	Stage I	171 (46.22%)	87 (47.03%)	84 (45.41%)	0.7738
	Stage II	85 (22.97%)	42 (22.7%)	43 (23.24%)	
	Stage III	85 (22.97%)	38 (20.54%)	47 (25.41%)	
	Stage IV	5 (1.35%)	3 (1.62%)	2 (1.08%)	
	Unknow	24 (6.49%)	15 (8.11%)	9 (4.86%)	

T	T1	181 (48.92%)	93 (50.27%)	88 (47.57%)	0.7336
	T2	93 (25.14%)	49 (26.49%)	44 (23.78%)	
	T3	80 (21.62%)	36 (19.46%)	44 (23.78%)	
	T4	13 (3.51%)	6 (3.24%)	7 (3.78%)	
	Unknow	3 (0.81%)	1 (0.54%)	2 (1.08%)	

M	M0	266 (71.89%)	137 (74.05%)	129 (69.73%)	1
	M1	4 (1.08%)	2 (1.08%)	2 (1.08%)	
	Unknow	100 (27.03%)	46 (24.86%)	54 (29.19%)	

N	N0	252 (68.11%)	120 (64.86%)	132 (71.35%)	0.5598
	N1	4 (1.08%)	3 (1.62%)	1 (0.54%)	
	Unknow	114 (30.81%)	62 (33.51%)	52 (28.11%)	

## Data Availability

The public data on HCC came from The Cancer Genome Atlas (TCGA) database, and data are available upon request to the authors.

## Supplementary Materials

Supplementary Materials
*Table S1*. Table of 15 CRGs and their cuproptosis death-related LncRNAs. *Table S2*. Table of KEGG pathway enrichment analysis of risk differential genes.
