# Supplementary material for: The Cuproptosis-Related Long Noncoding RNA Signature Predicts Prognosis and Immune Cell Infiltration in Hepatocellular Carcinoma
Source: J Oncol. 2023 Feb 27;2023:9557690. doi: 10.1155/2023/9557690 (PMC9988371; doi:10.1155/2023/9557690)
Supplement: Supplementary Materials — Table S1. Table of 15 CRGs and their cuproptosis death-related LncRNAs. Table S2. Table of KEGG pathway enrichment analysis of risk differential genes. [file 9557690.f1.doc]

***Supplementary Information For***

**The Cuproptosis-related long non-coding RNAs signature predicts prognosis and immune cell infiltration in hepatocellular carcinoma.**

Ying Li^1, 2†^, Kaichao Song^1†^, Wensheng Zheng^1^* (1. Beijing City Key Laboratory of Drug Delivery Technology and Novel Formulation, Institute of Materia Medica, Chinese Academy of Medical Sciences & Peking Union Medical College, Beijing 100050, China; 2. Shandong University of Traditional Chinese Medicine, College of Traditional Chinese Medicine, Jinan 250355, China)

***Correspondence:** Wensheng Zheng, Beijing Key Laboratory of Drug Delivery and Novel Formulation, Institute of Materia Medica, Chinese Academy of Medical Sciences & Peking Union Medical College, Beijing, China. E-mail: wensheng_zheng@126.com Phone/Fax: 0086-010-63165233.

^†^The author's contribution to the article is equal.

**Table S1 Table of 15 CRGs and their coproptosis death-related LncRNAs**

| **Cuproptosis** | **lncRNA** | **cor** | **P value** |
| --- | --- | --- | --- |
| ATP7A | CTBP1-DT | 0.516 | 8.53E-27 |
| ATP7A | AL606489.1 | 0.530 | 1.56E-28 |
| ATP7A | FAM111A-DT | 0.562 | 1.55E-32 |
| ATP7A | AL035411.3 | 0.546 | 2.02E-30 |
| ATP7A | NORAD | 0.571 | 1.09E-33 |
| ATP7A | AC114956.2 | 0.503 | 1.98E-25 |
| ATP7A | LINC00909 | 0.501 | 3.36E-25 |
| ATP7A | LINC00265 | 0.506 | 9.76E-26 |
| ATP7A | AC007406.4 | 0.561 | 2.15E-32 |
| ATP7A | LINC00294 | 0.613 | 6.58E-40 |
| ATP7A | AL109936.9 | 0.533 | 6.88E-29 |
| ATP7A | AC145098.1 | 0.524 | 9.43E-28 |
| ATP7A | LINC02035 | 0.550 | 6.23E-31 |
| ATP7A | AL122035.1 | 0.570 | 1.51E-33 |
| ATP7A | LINC01094 | 0.568 | 2.17E-33 |
| ATP7A | AC011462.5 | 0.559 | 3.79E-32 |
| ATP7A | AC120114.1 | 0.577 | 1.27E-34 |
| ATP7A | LINC01772 | 0.514 | 1.25E-26 |
| ATP7A | LINC00630 | 0.769 | 2.57E-74 |
| ATP7A | HCG18 | 0.523 | 1.24E-27 |
| ATP7A | ZNF32-AS2 | 0.515 | 1.05E-26 |
| ATP7A | AL592295.4 | 0.507 | 8.56E-26 |
| ATP7A | AC025171.2 | 0.541 | 7.36E-30 |
| ATP7A | LINC00205 | 0.529 | 2.13E-28 |
| ATP7A | AL360270.1 | 0.504 | 1.90E-25 |
| ATP7A | LINC01278 | 0.724 | 6.77E-62 |
| ATP7A | AC004067.1 | 0.511 | 2.77E-26 |
| ATP7A | AC092614.1 | 0.521 | 2.32E-27 |
| ATP7A | NRAV | 0.526 | 4.75E-28 |
| ATP7A | AC013486.1 | 0.529 | 2.31E-28 |
| ATP7A | AC073046.1 | 0.554 | 1.98E-31 |
| ATP7A | NNT-AS1 | 0.529 | 2.28E-28 |
| ATP7A | AL603839.2 | 0.516 | 6.98E-27 |
| ATP7A | MAP3K14-AS1 | 0.501 | 3.39E-25 |
| ATP7A | AC026979.4 | 0.544 | 3.68E-30 |
| ATP7A | AC004596.1 | 0.545 | 2.40E-30 |
| ATP7A | EBLN3P | 0.649 | 4.18E-46 |
| ATP7A | AL356481.1 | 0.526 | 5.23E-28 |
| ATP7A | ALKBH3-AS1 | 0.506 | 9.29E-26 |
| ATP7A | BTG3-AS1 | 0.606 | 8.31E-39 |
| ATP7A | AC010834.3 | 0.544 | 3.44E-30 |
| ATP7A | FGD5-AS1 | 0.624 | 8.65E-42 |
| ATP7A | AC006008.1 | 0.538 | 1.68E-29 |
| ATP7A | AC090559.1 | 0.514 | 1.17E-26 |
| ATP7A | AL590705.3 | 0.521 | 2.06E-27 |
| ATP7A | AC026356.1 | 0.563 | 1.22E-32 |
| ATP7A | AC005670.3 | 0.628 | 2.08E-42 |
| ATP7A | Z68871.1 | 0.659 | 7.23E-48 |
| ATP7A | RNF213-AS1 | 0.557 | 8.25E-32 |
| ATP7A | AC108463.2 | 0.557 | 8.01E-32 |
| ATP7A | AC022150.4 | 0.502 | 2.90E-25 |
| ATP7A | AC010186.3 | 0.557 | 6.64E-32 |
| ATP7A | LINC01560 | 0.524 | 8.90E-28 |
| ATP7A | SMARCA5-AS1 | 0.581 | 4.24E-35 |
| ATP7B | AC008764.2 | 0.502 | 2.90E-25 |

Note: All the samples’ Regulation is positive.

**Table S1: Table of 15 CRGs and their coproptosis death-related LncRNAs (Cont 1)**

| **Cuproptosis** | **lncRNA** | **cor** | **P value** |
| --- | --- | --- | --- |
| CDKN2A | MIR924HG | 0.507 | 8.15E-26 |
| CDKN2A | AC026401.3 | 0.628 | 1.84E-42 |
| CDKN2A | CDKN2B-AS1 | 0.737 | 3.22E-65 |
| CDKN2A | GIHCG | 0.514 | 1.28E-26 |
| CDKN2A | DDX11-AS1 | 0.576 | 2.21E-34 |
| CDKN2A | CDKN2A-DT | 0.649 | 4.07E-46 |

**Table S1: Table of 15 CRGs and their coproptosis death-related LncRNAs (Cont 2)**

| **Cuproptosis** | **lncRNA** | **cor** | **P value** |
| --- | --- | --- | --- |
| DBT | AC018647.2 | 0.537 | 2.52E-29 |
| DBT | DHRS4-AS1 | 0.513 | 1.70E-26 |
| DBT | PAXIP1-AS2 | 0.524 | 8.71E-28 |
| DBT | AC135050.5 | 0.510 | 4.04E-26 |
| DLAT | LINC02547 | 0.512 | 2.18E-26 |
| DLD | PAXIP1-AS2 | 0.585 | 1.00E-35 |

**Table S1: Table of 15 CRGs and their coproptosis death-related LncRNAs (Cont 3)**

| **Cuproptosis** | **lncRNA** | **cor** | **P value** |
| --- | --- | --- | --- |
| GLS | NIPBL-DT | 0.516 | 7.95E-27 |
| GLS | LINC01011 | 0.567 | 3.42E-33 |
| GLS | MIATNB | 0.624 | 8.90E-42 |
| GLS | AC087289.5 | 0.510 | 3.84E-26 |
| GLS | FAM111A-DT | 0.657 | 1.35E-47 |
| GLS | AC027702.1 | 0.525 | 7.79E-28 |
| GLS | AP001372.2 | 0.507 | 7.50E-26 |
| GLS | AC004812.2 | 0.510 | 3.53E-26 |
| GLS | BX322234.1 | 0.537 | 2.28E-29 |
| GLS | NORAD | 0.508 | 6.11E-26 |
| GLS | Z97989.1 | 0.542 | 5.51E-30 |
| GLS | AC018904.1 | 0.543 | 4.66E-30 |
| GLS | AC083855.2 | 0.571 | 1.01E-33 |
| GLS | LINC00265 | 0.553 | 2.25E-31 |
| GLS | AC027237.3 | 0.602 | 2.95E-38 |
| GLS | AC060766.4 | 0.571 | 8.38E-34 |
| GLS | AC019080.1 | 0.510 | 4.23E-26 |
| GLS | AC079781.5 | 0.512 | 2.41E-26 |
| GLS | UBE2Q1-AS1 | 0.504 | 1.81E-25 |
| GLS | GUSBP11 | 0.510 | 3.40E-26 |
| GLS | AC087294.1 | 0.510 | 3.80E-26 |
| GLS | LINC02035 | 0.571 | 9.37E-34 |
| GLS | AL122035.1 | 0.504 | 1.73E-25 |
| GLS | NUTM2B-AS1 | 0.527 | 4.08E-28 |
| GLS | AC008764.8 | 0.564 | 7.58E-33 |
| GLS | MIR4435-2HG | 0.652 | 1.06E-46 |
| GLS | AC060766.6 | 0.515 | 1.05E-26 |
| GLS | AC091057.1 | 0.505 | 1.33E-25 |
| GLS | AC026356.2 | 0.541 | 7.87E-30 |
| GLS | AC011462.5 | 0.575 | 2.72E-34 |
| GLS | LINC00342 | 0.588 | 3.37E-36 |
| GLS | AL358472.4 | 0.505 | 1.45E-25 |
| GLS | LINC00630 | 0.577 | 1.35E-34 |
| GLS | ZNF32-AS2 | 0.598 | 1.09E-37 |
| GLS | LINC00205 | 0.505 | 1.39E-25 |
| GLS | AC009120.2 | 0.509 | 4.89E-26 |
| GLS | Z92544.1 | 0.551 | 4.25E-31 |
| GLS | AC110611.1 | 0.504 | 1.81E-25 |
| GLS | FAM66C | 0.629 | 1.45E-42 |
| GLS | NRSN2-AS1 | 0.511 | 3.12E-26 |
| GLS | AL355388.1 | 0.555 | 1.52E-31 |
| GLS | AC004067.1 | 0.606 | 7.02E-39 |
| GLS | NRAV | 0.635 | 1.17E-43 |
| GLS | AP000759.1 | 0.511 | 2.92E-26 |
| GLS | CHROMR | 0.509 | 4.28E-26 |
| GLS | AC013486.1 | 0.509 | 4.34E-26 |
| GLS | Z69733.1 | 0.590 | 1.72E-36 |
| GLS | U52111.1 | 0.539 | 1.49E-29 |
| GLS | AC016065.1 | 0.520 | 2.76E-27 |
| GLS | AC005261.1 | 0.532 | 1.08E-28 |
| GLS | MAP3K14-AS1 | 0.568 | 2.79E-33 |
| GLS | AL731567.1 | 0.511 | 2.71E-26 |
| GLS | AC009093.1 | 0.501 | 3.84E-25 |
| GLS | AC061975.8 | 0.536 | 3.68E-29 |
| GLS | THAP9-AS1 | 0.501 | 3.81E-25 |
| GLS | AL353748.3 | 0.573 | 5.50E-34 |
| GLS | SCTR-AS1 | 0.515 | 1.09E-26 |
| GLS | DUBR | 0.586 | 6.24E-36 |
| GLS | AC026979.4 | 0.519 | 3.89E-27 |
| GLS | MRPS30-DT | 0.543 | 3.99E-30 |
| GLS | AL121772.3 | 0.511 | 2.88E-26 |
| GLS | HMGN3-AS1 | 0.530 | 1.86E-28 |
| GLS | BX842570.1 | 0.531 | 1.19E-28 |
| GLS | AC091185.1 | 0.528 | 2.94E-28 |
| GLS | ANKRD10-IT1 | 0.505 | 1.52E-25 |
| GLS | AL499602.1 | 0.516 | 8.63E-27 |
| GLS | AC009061.2 | 0.502 | 3.11E-25 |
| GLS | AC109587.1 | 0.549 | 8.46E-31 |
| GLS | AL078644.1 | 0.524 | 8.93E-28 |
| GLS | MCPH1-AS1 | 0.501 | 3.65E-25 |
| GLS | AC092953.2 | 0.520 | 2.45E-27 |
| GLS | AC006008.1 | 0.611 | 1.30E-39 |
| GLS | AF131215.5 | 0.573 | 4.79E-34 |
| GLS | YEATS2-AS1 | 0.520 | 2.93E-27 |
| GLS | GORAB-AS1 | 0.514 | 1.33E-26 |
| GLS | AC073130.2 | 0.536 | 3.53E-29 |
| GLS | AC016705.2 | 0.620 | 4.23E-41 |
| GLS | AC026356.1 | 0.551 | 3.93E-31 |
| GLS | AC060766.7 | 0.589 | 2.40E-36 |
| GLS | AC116351.1 | 0.583 | 2.33E-35 |
| GLS | AC004908.1 | 0.528 | 3.27E-28 |
| GLS | WARS2-AS1 | 0.523 | 1.06E-27 |
| GLS | AC008760.1 | 0.513 | 1.83E-26 |
| GLS | AL139407.1 | 0.511 | 2.74E-26 |
| GLS | AC108463.2 | 0.591 | 1.23E-36 |
| GLS | AL157392.3 | 0.543 | 5.24E-30 |

**Table S1: Table of 15 CRGs and their coproptosis death-related LncRNAs (Cont 4)**

| **Cuproptosis** | **lncRNA** | **cor** | **P value** |
| --- | --- | --- | --- |
| LIAS | CTD-2350J17.1 | 0.600 | 5.53E-38 |
| LIAS | AL450263.1 | 0.569 | 1.81E-33 |
| LIAS | PITPNM2-AS1 | 0.634 | 2.30E-43 |
| LIAS | AP003031.1 | 0.506 | 1.06E-25 |
| LIAS | AL161941.1 | 0.606 | 8.60E-39 |
| LIAS | AC008554.1 | 0.501 | 3.59E-25 |
| LIAS | AP001198.2 | 0.622 | 2.09E-41 |
| LIAS | AC009410.1 | 0.531 | 1.42E-28 |
| LIAS | TH2LCRR | 0.636 | 8.66E-44 |

**Table S1: Table of 15 CRGs and their coproptosis death-related LncRNAs (Cont 5)**

| **Cuproptosis** | **lncRNA** | **cor** | **P value** |
| --- | --- | --- | --- |
| LIPT1 | AL357079.1 | 0.560 | 3.02E-32 |
| LIPT1 | SPRY4-AS1 | 0.515 | 1.05E-26 |
| LIPT1 | LINC01011 | 0.564 | 9.66E-33 |
| LIPT1 | FAM111A-DT | 0.514 | 1.19E-26 |
| LIPT1 | AC004812.2 | 0.553 | 2.43E-31 |
| LIPT1 | AC005730.3 | 0.509 | 4.64E-26 |
| LIPT1 | AC096642.1 | 0.556 | 1.12E-31 |
| LIPT1 | AC009506.1 | 0.505 | 1.33E-25 |
| LIPT1 | AL162595.1 | 0.501 | 3.82E-25 |
| LIPT1 | AC022210.1 | 0.507 | 7.35E-26 |
| LIPT1 | AC019080.1 | 0.536 | 3.52E-29 |
| LIPT1 | STX18-AS1 | 0.510 | 3.92E-26 |
| LIPT1 | AC010245.2 | 0.500 | 4.39E-25 |
| LIPT1 | AC008764.8 | 0.535 | 5.02E-29 |
| LIPT1 | MIR4435-2HG | 0.611 | 1.16E-39 |
| LIPT1 | LINC00342 | 0.514 | 1.16E-26 |
| LIPT1 | AC097448.1 | 0.522 | 1.45E-27 |
| LIPT1 | AC023302.1 | 0.507 | 8.29E-26 |
| LIPT1 | AL358472.4 | 0.526 | 4.99E-28 |
| LIPT1 | EIF2AK3-DT | 0.510 | 3.53E-26 |
| LIPT1 | HCG18 | 0.512 | 2.27E-26 |
| LIPT1 | AL731533.2 | 0.520 | 2.85E-27 |
| LIPT1 | AC016747.1 | 0.500 | 4.55E-25 |
| LIPT1 | SEC24B-AS1 | 0.532 | 9.56E-29 |
| LIPT1 | LINC00653 | 0.553 | 2.46E-31 |
| LIPT1 | AC009120.2 | 0.528 | 3.27E-28 |
| LIPT1 | ZEB1-AS1 | 0.533 | 7.74E-29 |
| LIPT1 | FAM66C | 0.506 | 9.18E-26 |
| LIPT1 | AC012360.3 | 0.567 | 3.38E-33 |
| LIPT1 | EIF3J-DT | 0.563 | 1.34E-32 |
| LIPT1 | AC126118.1 | 0.501 | 3.33E-25 |
| LIPT1 | STARD7-AS1 | 0.531 | 1.39E-28 |
| LIPT1 | NIFK-AS1 | 0.601 | 4.51E-38 |
| LIPT1 | NRAV | 0.524 | 9.59E-28 |
| LIPT1 | WAC-AS1 | 0.598 | 1.28E-37 |
| LIPT1 | AC017083.1 | 0.521 | 1.81E-27 |
| LIPT1 | CAPN10-DT | 0.591 | 1.47E-36 |
| LIPT1 | THUMPD3-AS1 | 0.532 | 9.67E-29 |
| LIPT1 | AC016065.1 | 0.516 | 7.64E-27 |
| LIPT1 | UBA6-AS1 | 0.532 | 1.17E-28 |
| LIPT1 | SCAMP1-AS1 | 0.545 | 2.58E-30 |
| LIPT1 | Z97832.2 | 0.521 | 2.31E-27 |
| LIPT1 | THAP9-AS1 | 0.516 | 8.00E-27 |
| LIPT1 | DUBR | 0.519 | 3.88E-27 |
| LIPT1 | MRPS30-DT | 0.548 | 9.31E-31 |
| LIPT1 | ASH1L-AS1 | 0.567 | 3.42E-33 |
| LIPT1 | SAP30L-AS1 | 0.538 | 2.12E-29 |
| LIPT1 | AC011462.4 | 0.562 | 1.76E-32 |
| LIPT1 | AC009061.2 | 0.599 | 8.62E-38 |
| LIPT1 | SNHG4 | 0.565 | 5.69E-33 |
| LIPT1 | AC092667.1 | 0.525 | 7.35E-28 |
| LIPT1 | AC010864.1 | 0.566 | 5.08E-33 |
| LIPT1 | AC116407.2 | 0.524 | 8.31E-28 |
| LIPT1 | AC021851.1 | 0.503 | 2.37E-25 |
| LIPT1 | AL359878.1 | 0.546 | 2.16E-30 |
| LIPT1 | LINC-PINT | 0.545 | 2.49E-30 |
| LIPT1 | TBC1D8-AS1 | 0.530 | 1.86E-28 |
| LIPT1 | NCK1-DT | 0.584 | 1.29E-35 |
| LIPT1 | LENG8-AS1 | 0.504 | 1.80E-25 |
| LIPT2 | AP001372.2 | 0.586 | 7.01E-36 |
| LIPT2 | ARIH2OS | 0.506 | 1.05E-25 |
| LIPT2 | BACE1-AS | 0.539 | 1.31E-29 |
| LIPT2 | LINC00205 | 0.552 | 3.09E-31 |
| LIPT2 | SNHG1 | 0.515 | 9.48E-27 |
| LIPT2 | AP000873.2 | 0.576 | 2.18E-34 |
| LIPT2 | RAB30-DT | 0.501 | 3.34E-25 |

**Table S1: Table of 15 CRGs and their coproptosis death-related LncRNAs (Cont 6)**

| **Cuproptosis** | **lncRNA** | **cor** | **P value** |
| --- | --- | --- | --- |
| MTF1 | CTBP1-DT | 0.522 | 1.44E-27 |
| MTF1 | FAM111A-DT | 0.531 | 1.27E-28 |
| MTF1 | AP001372.2 | 0.503 | 2.30E-25 |
| MTF1 | AL109627.1 | 0.524 | 9.04E-28 |
| MTF1 | AL035411.3 | 0.553 | 2.39E-31 |
| MTF1 | AL031282.2 | 0.523 | 1.07E-27 |
| MTF1 | NORAD | 0.539 | 1.41E-29 |
| MTF1 | AC026412.3 | 0.520 | 2.77E-27 |
| MTF1 | AC007406.4 | 0.513 | 1.83E-26 |
| MTF1 | AC019080.1 | 0.511 | 3.01E-26 |
| MTF1 | AC102953.2 | 0.501 | 4.10E-25 |
| MTF1 | AC005034.5 | 0.513 | 1.85E-26 |
| MTF1 | LINC02035 | 0.516 | 8.74E-27 |
| MTF1 | AC007390.1 | 0.510 | 3.43E-26 |
| MTF1 | AC120114.1 | 0.508 | 6.36E-26 |
| MTF1 | LINC01772 | 0.516 | 7.71E-27 |
| MTF1 | LINC00630 | 0.604 | 1.66E-38 |
| MTF1 | PAXIP1-AS2 | 0.556 | 9.80E-32 |
| MTF1 | LINC00205 | 0.525 | 6.55E-28 |
| MTF1 | NRSN2-AS1 | 0.511 | 2.61E-26 |
| MTF1 | STARD7-AS1 | 0.505 | 1.51E-25 |
| MTF1 | NRAV | 0.524 | 8.37E-28 |
| MTF1 | AC004596.1 | 0.516 | 8.32E-27 |
| MTF1 | EBLN3P | 0.578 | 9.93E-35 |
| MTF1 | AL031985.3 | 0.507 | 8.20E-26 |
| MTF1 | AC073254.1 | 0.502 | 2.53E-25 |
| MTF1 | AC000123.1 | 0.534 | 5.57E-29 |
| MTF1 | FGD5-AS1 | 0.568 | 2.83E-33 |
| MTF1 | YEATS2-AS1 | 0.508 | 5.85E-26 |
| MTF1 | AC098484.4 | 0.537 | 2.67E-29 |
| MTF1 | AC005670.3 | 0.608 | 3.42E-39 |
| MTF1 | Z68871.1 | 0.574 | 3.40E-34 |
| MTF1 | WARS2-AS1 | 0.602 | 3.30E-38 |
| MTF1 | NCK1-DT | 0.520 | 2.55E-27 |
| MTF1 | RNF213-AS1 | 0.591 | 1.16E-36 |
| MTF1 | SMARCA5-AS1 | 0.556 | 1.01E-31 |

**Table S1: Table of 15 CRGs and their coproptosis death-related LncRNAs (Cont 7)**

| **Cuproptosis** | **lncRNA** | **cor** | **P value** |
| --- | --- | --- | --- |
| NFE2L2 | AL132800.1 | 0.507 | 8.03E-26 |
| NFE2L2 | AC007406.4 | 0.515 | 9.61E-27 |
| NFE2L2 | AC019080.1 | 0.549 | 9.17E-31 |
| NFE2L2 | AC097448.1 | 0.508 | 6.66E-26 |
| NFE2L2 | PAXIP1-AS2 | 0.520 | 2.98E-27 |
| NFE2L2 | USP46-DT | 0.536 | 3.33E-29 |
| NFE2L2 | EBLN3P | 0.585 | 8.85E-36 |
| NFE2L2 | AC073254.1 | 0.570 | 1.44E-33 |
| NFE2L2 | FGD5-AS1 | 0.603 | 1.83E-38 |

**Table S1: Table of 15 CRGs and their coproptosis death-related LncRNAs (Cont 8)**

| **Cuproptosis** | **lncRNA** | **cor** | **P value** |
| --- | --- | --- | --- |
| NLRP3 | TFAP2E-AS1 | 0.642 | 7.76E-45 |
| NLRP3 | AC006272.1 | 0.537 | 2.74E-29 |
| NLRP3 | LINC02345 | 0.578 | 1.10E-34 |
| NLRP3 | AC002091.2 | 0.707 | 5.73E-58 |
| NLRP3 | HOTAIRM1 | 0.504 | 1.89E-25 |
| NLRP3 | BX322234.1 | 0.538 | 2.18E-29 |
| NLRP3 | AC007877.1 | 0.576 | 1.78E-34 |
| NLRP3 | AC108134.3 | 0.623 | 1.36E-41 |
| NLRP3 | LINC01943 | 0.542 | 6.35E-30 |
| NLRP3 | LINC00996 | 0.510 | 3.35E-26 |
| NLRP3 | AP002954.1 | 0.643 | 4.55E-45 |
| NLRP3 | L3MBTL4-AS1 | 0.668 | 1.20E-49 |
| NLRP3 | C9orf139 | 0.706 | 9.43E-58 |
| NLRP3 | AC138207.5 | 0.789 | 1.52E-80 |
| NLRP3 | DNM3OS | 0.540 | 9.67E-30 |
| NLRP3 | AC008105.3 | 0.553 | 2.36E-31 |
| NLRP3 | AC069360.1 | 0.505 | 1.29E-25 |
| NLRP3 | AC004585.1 | 0.511 | 3.23E-26 |
| NLRP3 | LINC01550 | 0.510 | 3.83E-26 |
| NLRP3 | LINC02723 | 0.502 | 2.64E-25 |
| NLRP3 | LINC02528 | 0.641 | 1.05E-44 |
| NLRP3 | AC023825.2 | 0.525 | 6.61E-28 |
| NLRP3 | AC145098.1 | 0.707 | 6.34E-58 |
| NLRP3 | PELATON | 0.505 | 1.24E-25 |
| NLRP3 | AL121933.2 | 0.613 | 6.18E-40 |
| NLRP3 | HIF1A-AS1 | 0.552 | 3.22E-31 |
| NLRP3 | LINC01679 | 0.659 | 5.81E-48 |
| NLRP3 | LINC01094 | 0.720 | 4.49E-61 |
| NLRP3 | AC110995.1 | 0.708 | 3.51E-58 |
| NLRP3 | PCED1B-AS1 | 0.661 | 2.47E-48 |
| NLRP3 | LINC01638 | 0.597 | 2.02E-37 |
| NLRP3 | PICSAR | 0.558 | 5.54E-32 |
| NLRP3 | ITGB2-AS1 | 0.587 | 5.23E-36 |
| NLRP3 | MIR155HG | 0.629 | 1.19E-42 |
| NLRP3 | CEP250-AS1 | 0.542 | 5.54E-30 |
| NLRP3 | AF127936.1 | 0.574 | 4.03E-34 |
| NLRP3 | AC015819.1 | 0.537 | 2.89E-29 |
| NLRP3 | CHROMR | 0.521 | 1.84E-27 |
| NLRP3 | AC013486.1 | 0.581 | 4.45E-35 |
| NLRP3 | AC021188.1 | 0.558 | 6.17E-32 |
| NLRP3 | LINC00539 | 0.653 | 6.85E-47 |
| NLRP3 | AC026369.3 | 0.501 | 3.95E-25 |
| NLRP3 | AL096865.1 | 0.553 | 2.78E-31 |
| NLRP3 | AC109479.1 | 0.526 | 5.06E-28 |
| NLRP3 | LINC02391 | 0.722 | 1.50E-61 |
| NLRP3 | AC000403.1 | 0.641 | 1.08E-44 |
| NLRP3 | LINC00702 | 0.562 | 1.53E-32 |
| NLRP3 | LINC00426 | 0.544 | 3.21E-30 |
| NLRP3 | AC138207.4 | 0.761 | 6.37E-72 |
| NLRP3 | AC009093.1 | 0.525 | 6.31E-28 |
| NLRP3 | LINC01711 | 0.577 | 1.62E-34 |
| NLRP3 | AC002091.1 | 0.733 | 3.45E-64 |
| NLRP3 | AC008759.3 | 0.552 | 3.46E-31 |
| NLRP3 | AC110611.2 | 0.581 | 3.27E-35 |
| NLRP3 | AC019254.1 | 0.512 | 2.16E-26 |
| NLRP3 | AC104530.1 | 0.650 | 2.38E-46 |
| NLRP3 | LINC02611 | 0.738 | 1.33E-65 |
| NLRP3 | AC090559.1 | 0.909 | 1.21E-143 |
| NLRP3 | MACORIS | 0.525 | 7.71E-28 |
| NLRP3 | HOMER3-AS1 | 0.553 | 2.11E-31 |
| NLRP3 | LINC01150 | 0.751 | 5.71E-69 |
| PDHA1 | NRSN2-AS1 | 0.538 | 1.81E-29 |
| SLC31A1 | LINC00261 | 0.505 | 1.25E-25 |

Table S2 Table of KEGG pathway enrichment analysis of risk differential genes

| ID | Description |
| --- | --- |
| hsa04060 | Cytokine-cytokine receptor interaction |
| hsa04640 | Hematopoietic cell lineage |
| hsa04061 | Viral protein interaction with cytokine and cytokine receptor |
| hsa05340 | Primary immunodeficiency |
| hsa04658 | Th1 and Th2 cell differentiation |
| hsa04514 | Cell adhesion molecules |
| hsa04659 | Th17 cell differentiation |
| hsa05330 | Allograft rejection |
| hsa04062 | Chemokine signaling pathway |
| hsa05320 | Autoimmune thyroid disease |
| hsa04660 | T cell receptor signaling pathway |
| hsa05332 | Graft-versus-host disease |
| hsa04940 | Type I diabetes mellitus |
| hsa04650 | Natural killer cell mediated cytotoxicity |
| hsa05416 | Viral myocarditis |
| hsa05166 | Human T-cell leukemia virus 1 infection |
| hsa05310 | Asthma |
| hsa05162 | Measles |
| hsa05169 | Epstein-Barr virus infection |
| hsa04064 | NF-kappa B signaling pathway |
| hsa05235 | PD-L1 expression and PD-1 checkpoint pathway in cancer |
